# Hornet’s Nest

**Published:** 1848-08

**Authors:** 


*Hornet's Nest.*—Dr. T. T. Lockwood of this city, at a late meeting of the Buffalo Medical Association, stated, that, when practising in the country, he had frequently prescribed *hornet's nest,* as an antispasmodic, and that particularly in whooping cough, he had found it to exert more influence in controlling the paroxysms and shortening the duration of the affection, than any other remedy he had ever tried. We suggest the employment of this article, especially to our brethren in the country, where the article can be readily procured, in order that its therapeutical value may be more fully tested. Will some of our classical friends invent a classical name by which to distinguish it, in season for its formal induction into the Materia Medica?—*Buffalo Medical Journal.*

